# Bibliography

**Published:** 1907-09-15

**Authors:** 


### BIBLIOGRAPHY.

DENTAL FORMULARY:—This is a work of some 300 pages devoted to the compounding of drugs for dental uses; embracing not only a great variety of useful technical formula, but therapeutic prescribing, also. The author, Dr. Herman Prinz, who is editor of the *Dental Era*, is well qualified by training and practice to undertake such a work. We are quite surprised in looking over the work to notice how few of the old and stereotyped formula have been incorporated in the book. The author takes up the formula in good order, beginning with Plaster of Paris and the various investment compounds; then the semi hard or plastic impression materials; the cements, varnishes etc.; metals and alloys; plating and lacquering; mouth toilet preparations; newer dental remedies; compounding and prescription writing; local anesthesia and anesthetics; and an index of oral diseases; closing the book with a large number of miscellaneous formula useful to the dentist. It also contains a number of blank pages for the collection of useful data and formula from ones reading or experience. The author is to be commended for the very satisfactory manner in which he has done the work and the profession should appreciate the great labor involved. Every dentist should have a copy of this book handy for reference and to enable him to record his own experiments. The book is published by the author, at St. Louis, Mo. We most cordially commend it to every practitioner as a very valuable work. The cost is three dollars and may be had of Jno. T. Nolde Co., 916 Oliver Street St. Louis, Mo.

The proceedings of the National Dental Society and its Southern Branch have just come to hand, almost one year after the meeting at Atlanta, Ga., last year. The book as usual is well printed but we do not like the change of the matter from one column on each page to two, and the enlargement of the page, it makes it awkward to shelve it in the library with former volumes which had a smaller page. It is printed by the *Dental Cosmos*, and that accounts for the change.

